# Primary Hydatid Cyst of the Right Maxillary Sinus: A Case Report

**Published:** 2014-10

**Authors:** Ali Reza Lotfi, Sina Zarrintan, Masoud Naderpour, Mohammad Sokhandan, Ashraf Fakhrjou, Amrollah Bayat, Firouz Salehpour, Hamid Djalilian

**Affiliations:** 1 *Department of Otorhinolaryngology, Imam Hospital, Tabriz University of Medical Sciences, Tabriz, Iran.*; 2 *Department of General and Vascular Surgery, Imam Reza Hospital, Tabriz University of Medical Sciences, Tabriz, Iran.*; 3 *Department of Pathology, Imam Hospital, Tabriz University of Medical Sciences, Tabriz, Iran.*; 4 *Department of Neurosurgery, Imam Hospital, Tabriz University of Medical Sciences, Tabriz, Iran.*; 5 **Department of Otolaryngology-Head and Neck Surgery, University of California, Irvine, Orange, CA, USA.**

**Keywords:** Cystic lesion, Echinococcus, Hydatid cyst, Maxillary sinus, Maxillofacial

## Abstract

**Introduction:**

A hydatid cyst of the head and neck is a very rare condition, even in areas where *Echinococcus* infestation is endemic.

**Case Report:**

We report a rare case of primary hydatid cyst of the right maxillary sinus in a 40-year-old man. The initial diagnosis of the presence of a cystic mass was the result of physical examination and computed tomography (CT) scan. We resected the cystic mass using the Caldwell-Luc procedure. A definitive diagnosis was confirmed by postoperative histopathologic examination.

**Conclusion:**

Hydatid cyst of the maxillary sinus is an extremely rare presentation. However, this condition should be considered in differential diagnosis of cystic lesions of the maxillary sinus.

## Introduction

Hydatid disease (also known as hydatidosis) is usually caused by the cestode *Echinococcus granulosus(E. granulosus)*, for which humans are an intermediate host (1,2). The main host and source of infection for humans is the dog. The infection is transferred to humans from unwashed hands after having touched a dog that has swallowed tapeworm eggs, from drinking water contaminated with dog feces, or by consuming fresh fruit and vegetables washed or irrigated with contaminated water (3). 

Hydatid disease occurs throughout the world and is especially common in sheep- and cattle-raising regions of Africa, Australia, New Zealand, India, the Middle East, South America, and the Mediterranean. The incidence of humans infected with hydatid disease is approximately 1–2:1,000, although it may be higher in rural areas of regions that are affected (4). It mainly affects the pulmonary and digestive systems. The liver is the most frequently involved organ (75%), followed by the lung (15%) and the rest of the body (10%) (5). 

The occurrence of hydatid cysts in the head and neck is rare, even in countries where *Echinococcus* infestation is endemic (6). Furthermore, hydatid cyst of the maxillofacial region has rarely been reported in the literature (2,4). Herein, we report a rare case of a hydatid cyst located in the right maxillary sinus. A broad review of the literature identified few cases of hydatid cyst of maxillary sinuses (2,4,7-11). 

## Case Report

**Fig 1:**
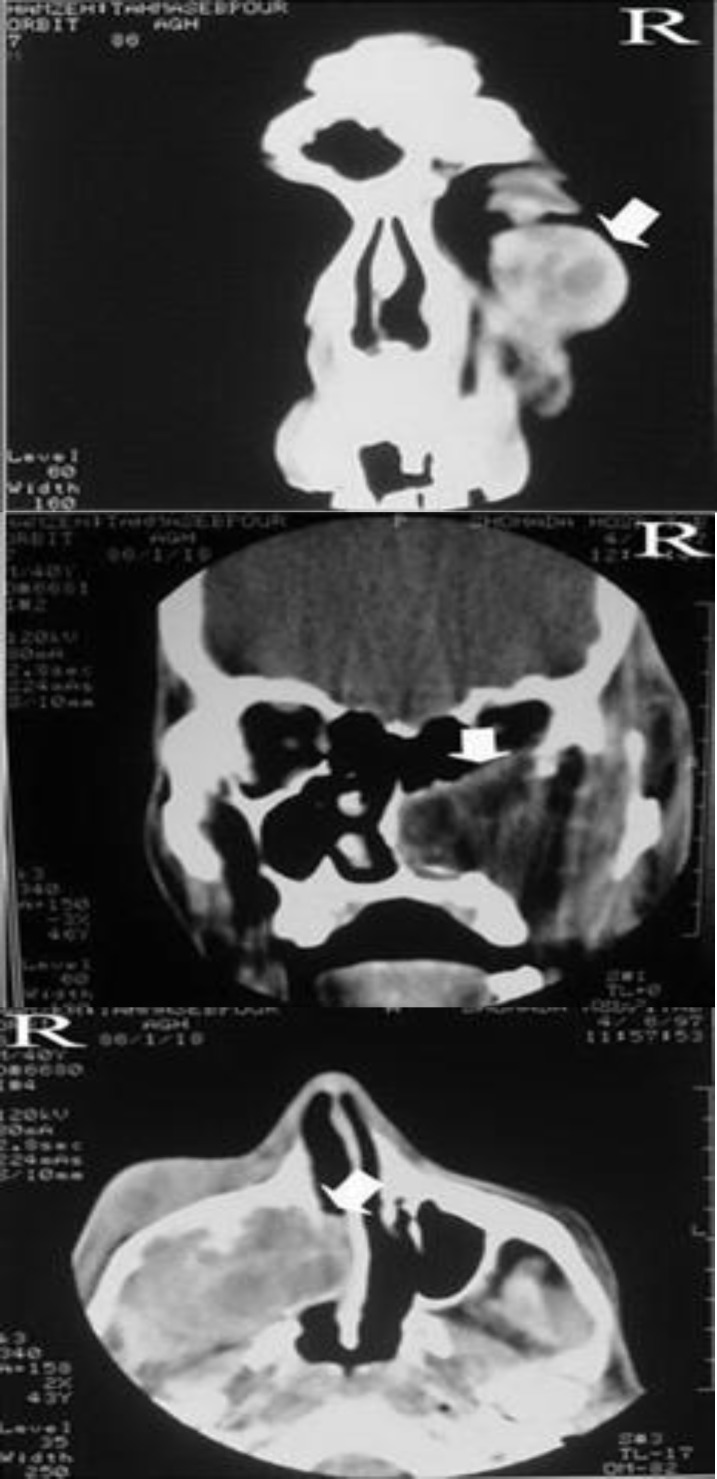
Coronal and axial CT sections of the head illustrating an increased density in the right maxillary sinus (arrows) together with erosions of its lateral, medial, and superior walls. R: Right side

A 40-year-old man with a painless swollen right cheek and right eyelid was referred to our department. The swelling had progressed gradually over the course of at least1 month and the patient had shown no improvement despite antibiotic treatment. The patient had clear rhinorrhea and a history of close contact with sheep and dogs. Physical examination revealed a well-shaped, firm, and fluctuant swelling with subcutaneous penetration without any evidence of inflammation. There was no tenderness in the affected area. Anterior rhinoscopy revealed lateral bulging of the right nasal wall. The general condition of the patient was otherwise normal. Computed tomography imaging (CT) of the face showed a loculated soft tissue density in the right maxillary sinus with erosion of the lateral and medial walls, as well as the floor of the orbit. The soft tissue density involved the orbit and compressed the globe (Fig.1). The appearance of the suspected mass by CT was suggestive of a cystic mass. Fine-needle aspiration (FNA) was performed, but this failed to provide sufficient material for fluid analysis. Given the compression of the orbit, we decided to resect the mass. 

**Fig 2:**
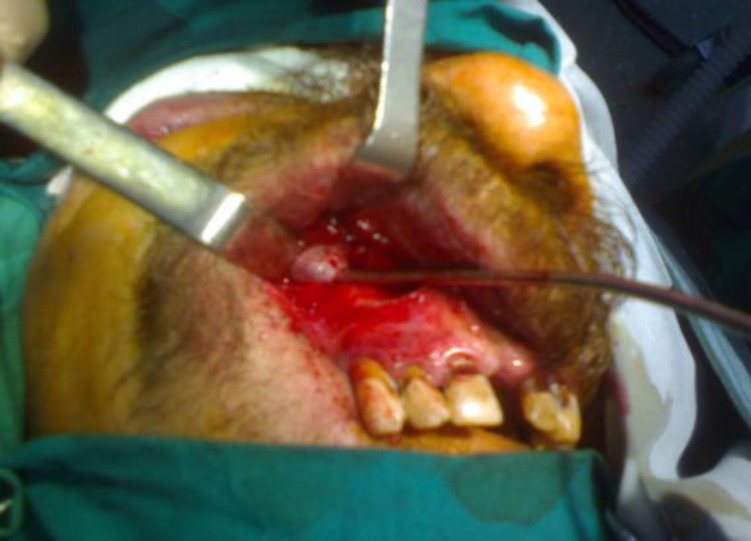
Caldwell-Luc procedure

A Caldwell-Luc approach was adopted for excision of the cystic mass (Fig. 2).

**Fig 3:**
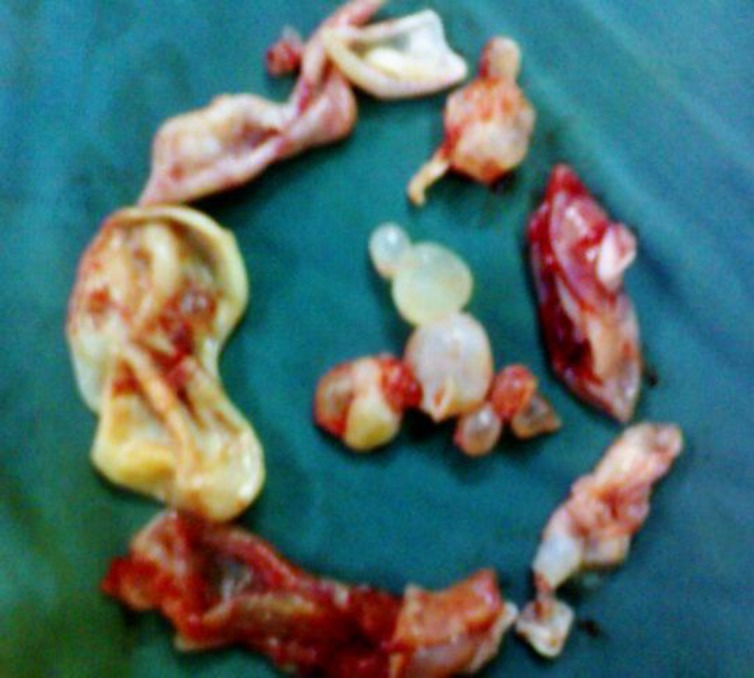
Macroscopic appearance of the hydatid cyst extracted from the right maxillary sinus

The mass in the maxillary sinus was yellow-white in color and elastic in nature, suggesting parasitic disease. We carefully removed the cystic mass and irrigated the sinus with hypertonic saline a total of three times. The macroscopic appearance of the extracted mass suggested a hydatid cyst. The cyst was enucleated easily, and multiple daughter cysts were found inside the capsule (Fig.3). 

**Fig 4:**
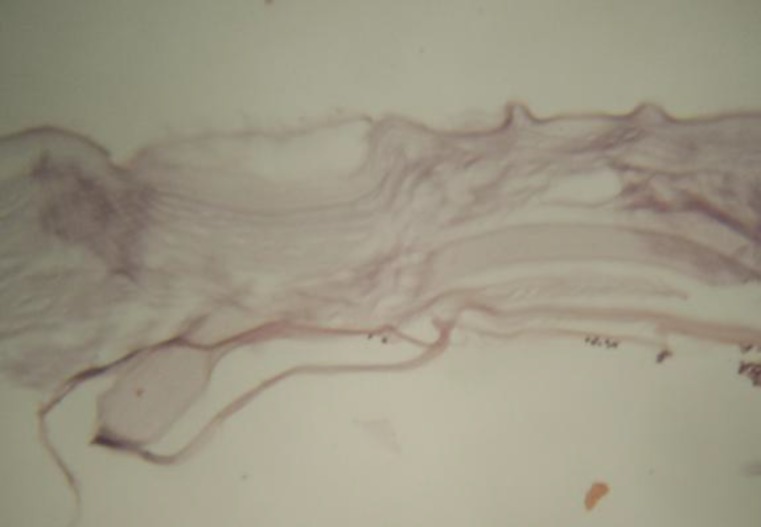
Light micrograph of the cystic material illustrating hydatid cyst wall (×400) stained by hematoxylin and eosin. The arrow illustrates the germinal layer

Histopathologic examination revealed a hydatid cyst consistent with *E. granulosus* (Fig.4). A serologic test of the hydatid cyst revealed a positive 1/264 result, which further confirmed the presence of hydatid disease.

An abdominal ultrasound and CT scan of the abdomen and thorax ruled out visceral or pulmonary involvement in the hydatid disease, which confirmed the diagnosis of a primary hydatid cyst of the right maxillary sinus. The patient was in a healthy condition after the operation and the swelling of the right cheek and surrounding areas were resolved rapidly. A 2-month course of albendazole (800 mg/d) with a 2-week interval after the first month was prescribed to the patient for prophylaxis against recurrence. The patient did not show any evidence of recurrence at follow-up 12 months after the operation.

## Discussion

Primary hydatid cysts of the maxillofacial region are relatively rare. Of the reported cases in the current literature review, only a few have involved the maxillary sinus (2,4,7-11). The first report of a probable hydatid cyst in the maxillary sinus was by Bohigues Sapena in 1969 (7). Since then only six other cases have been reported in the literature (2,4,8-11). Including the present case, there have been just eight cases of maxillary sinus hydatidosis reported.

Most hydatid cysts in the head and neck are asymptomatic, with symptoms depending on the location and size of the cyst. Lesions are characteristically slow growing (6,12). A complete history of the patient’s occupation, residence, and exposure to specific animals or materials play a key role in clinical suspicion. Although the history can raise the possibility of hydatid disease in the differential diagnosis of head and neck cystic masses, preoperative diagnosis may be missed without demonstrative radiologic findings (6). 

Plain radiography, ultrasound, CT, and magnetic resonance imaging (MRI) are the most useful diagnostic tools for detection of hydatid cystic lesions of the maxillofacial region (2). CT and MRI in particular are highly sensitive for the diagnosis of hydatidosis and provide a complete lesion work-up (2,13). Serologic tests such as direct hemagglutination, latex agglutination, and immunoelectrophoresis are widely used to confirm the diagnosis; however, they have low sensitivity and specificity. Serologic tests may also remain positive months to years after treatment, and thus have a limited role in patient follow-up and detection of relapse (6,14). 

Besides the excellent diagnostic value of imaging techniques in the detection of hydatid cysts, it is sometimes difficult to determine the cystic nature of a radiologically-visualized mass (2).

In addition, it is sometimes difficult to distinguish a hydatid cystic lesion from a simple cystic structure (6). This is a potential shortcoming of imaging techniques in the diagnosis of hydatid cysts which may be hazardous. Cystic visualization of the suspected mass may persuade the clinician to conduct FNA, which is a controversial procedure for hydatid cysts (6,15). This controversy is due to the potential to precipitate acute anaphylaxis or to spread daughter cysts (6,14-16). In the case report by Ataoglu et al. (2), a mass was visualized in the right maxillary sinus by CT imaging. The authors subsequently conducted a FNA, which recovered clear cystic fluid under the presumed diagnosis of a mucocele or dentigerous cyst. It is important for the clinician to consider hydatid cysts in the maxillary sinus and other areas of the head and neck in the at-risk population before conducting FNA in order to avoid potential associated risks. 

We also visualized a loculated mass in the right maxillary sinus in the present case, which was suggestive of a cystic structure. In our case, the diagnosis was made postoperatively through histopathologic examination. A hydatid cyst was not considered in our preoperative differential diagnosis. FNA did not cause an anaphylactic reaction or spreading of the cyst. Thus, a hydatid cyst should be considered in the differential diagnosis of cystic masses of the head and neck in, and FNA should be avoided unless hydatidosis is ruled out.

Surgery is generally the treatment of choice for hydatid disease unless resection is not possible (for example in cases of multiple organ involvement, inaccessible location, or poor general health) (6,17). During the operation, the cyst should be removed with the germinative layer, carefully avoiding the spillage of the cystic contents. After excision of the cyst, the potential space can then be obliterated. This technique has been termed “cystotomy and capitonnage” (6,12). Ataoglu et al. (2) surgically explored the site of the cystic mass, removed the cyst, and then irrigated the surgical area with hypertonic saline in their case of right maxillary hydatidosis. We also explored the right maxillary sinus through the Caldwell-Luc approach and extracted the cystic mass. We recommend the Caldwell-Luc approach for surgical resection of maxillary sinus hydatid disease. This approach allows removal of the cyst without spillage to ensure total excision. Irrigation of the surgical area with hypertonic saline is also recommended after removal of the cyst to lyse any remaining parasites in the area.

Combination of surgical therapy with imidazole derivatives has been used for the prevention of recurrences (2,6). Although there were no signs of abdominal or pulmonary involvement in the present case, we prescribed a 2-month course of albendazole (800 mg/d) to the patient. In addition to prophylaxis against recurrence, medical therapy may also be a substitute for surgery when surgery is not possible (6). Percutaneous treatment of the hydatid cyst has also been suggested as another alternative to surgery, but may not be preferred in the treatment of maxillofacial hydatid cyst if there is intervening bone (6,18). 

## Conclusion

Although maxillary sinus is an extremely rare location for a hydatid cyst, this possibility should be considered in the differential diagnosis of a maxillofacial mass. Imaging techniques are highly beneficial in the primary diagnosis; however, the definitive diagnosis is usually made by postoperative histopathologic examinations. The principal treatment of maxillary sinus hydatidosis is surgery and postoperative medical treatment to prevent recurrence. FNA carries the risk of anaphylactic reactions and it should be avoided when the diagnosis of hydatid cyst is considered.
